# Biocrusts from Iceland and Svalbard: Does microbial community composition differ substantially?

**DOI:** 10.3389/fmicb.2022.1048522

**Published:** 2022-12-16

**Authors:** Ekaterina Pushkareva, Josef Elster, Andreas Holzinger, Sarina Niedzwiedz, Burkhard Becker

**Affiliations:** ^1^Department of Biology, Botanical Institute, University of Cologne, Cologne, Germany; ^2^Institute of Botany, Academy of Sciences of the Czech Republic, Trebon, Czechia; ^3^Centre for Polar Ecology, University of South Bohemia, Ceske Budejovice, Czechia; ^4^Functional Plant Biology, Department of Botany, University of Innsbruck, Innsbruck, Austria; ^5^Marine Botany, Faculty of Biology and Chemistry & MARUM, University of Bremen, Bremen, Germany

**Keywords:** biocrusts, bacteria, eukaryotes, microbial phototrophs, amplicon sequencing, co-occurrence

## Abstract

A wide range of microorganisms inhabit biocrusts of arctic and sub-arctic regions. These taxa live and thrive under extreme conditions and, moreover, play important roles in biogeochemical cycling. Nevertheless, their diversity and abundance remain ambiguous. Here, we studied microbial community composition in biocrusts from Svalbard and Iceland using amplicon sequencing and epifluorescence microscopy. Sequencing of 16S rRNA gene revealed the dominance of Chloroflexi in the biocrusts from Iceland and Longyearbyen, and Acidobacteria in the biocrusts from Ny-Ålesund and South Svalbard. Within the 18S rRNA gene sequencing dataset, Chloroplastida prevailed in all the samples with dominance of Trebouxiophyceae in the biocrusts from Ny-Ålesund and Embryophyta in the biocrusts from the other localities. Furthermore, cyanobacterial number of cells and biovolume exceeded the microalgal in the biocrusts. Community compositions in the studied sites were correlated to the measured chemical parameters such as conductivity, pH, soil organic matter and mineral nitrogen contents. In addition, co-occurrence analysis showed the dominance of positive potential interactions and, bacterial and eukaryotic taxa co-occurred more frequently together.

## Introduction

Biocrusts (biological soil crusts) cover large ice-free territories in polar and sub-polar regions. They are associations of prokaryotic and eukaryotic microbial communities living on the uppermost soil surface. Biocrusts are usually around 5 mm thick and contain organisms, which produce extracellular polymeric substances and possess filamentous structures (Weber et al., 2022). These increase the stability of the soil surface and reduce the effect of erosion. Furthermore, biocrusts contribute to the carbon (C) and nutrient cycles, and play an important role in partially vegetated areas promoting growth and development of vascular plants and small animal communities (Bowker et al., 2010; Barrera et al., 2022).

Biocrusts in the northern latitudes undergo frequent disturbances (e.g., water-wind erosion, soil cryo-disturbance, anthropogenic activities, animal grazing, etc.), which greatly influence microbial biodiversity and abundance. Besides, soil parent material and organic matter (SOM) content also determine microbial community composition and, subsequently, the biocrust development stage (Pushkareva et al., 2016). Biocrusts are extremotolerant and, thus, can endure low temperatures and long periods of desiccation (Schmidt and Vimercati, 2019). Moreover, some biocrust microorganisms produce pigments, which serve as sunscreen against UV radiation (Karsten and Holzinger, 2014).

The Arctic region lies north of the tree line and is characterized as a polar desert and/or semi-desert. It has a limited number of vascular plants and biocrusts are the main type of vegetation there (Williams et al., 2016). Several studies showed that Arctic biocrusts are dominated by photoautotrophic microorganisms, such as cyanobacteria and microalgae (Pushkareva et al., 2016; Rippin et al., 2018a). The most abundant cyanobacteria in this environment are filamentous forms from orders Pseudanabaenales and Oscillatoriales and heterocyst-forming order Nostocales. Microalgal communities in arctic biocrusts are mainly represented by Chlorophyceae and Xanthophyceae as well as Bacillariophyceae.

Iceland with oceanic subarctic climate is located right below the Arctic circle. The soils of Iceland (Andosols) are of volcanic origin with high organic matter content and very distinct from the arctic soils (Arnalds, 2008). The biocrust community composition is mainly represented by Proteobacteria within prokaryotes and by green algae within eukaryotes (Pushkareva et al., 2021a). Diatoms are also very abundant in Icelandic biocrust (Pushkareva et al., 2021b).

Despite the studies describing biodiversity of arctic and sub-arctic biocrusts, the knowledge about their community composition remains incomplete. The distribution of microorganisms and their abundance is of crucial importance for understanding recent and ancient environmental dynamics. In this regard, we studied prokaryotic and eukaryotic microbiota in biocrusts from Svalbard (Ny-Ålesund and Longyearbyen areas, and south of Svalbard) and Iceland using molecular and morphological approaches. Svalbard and Iceland are washed by the Gulf Stream and share some flora and fauna together. Moreover, climate change effect is more pronounced in these islands than in the northern European countries. We hypothesized that differences in (1) latitude and (2) soil chemistry would influence biocrust community composition. We expected that microbial diversity would decrease with increasing the latitude due to the more severe environmental conditions there.

## Materials and methods

### Site description and sampling

**Figure 1:**
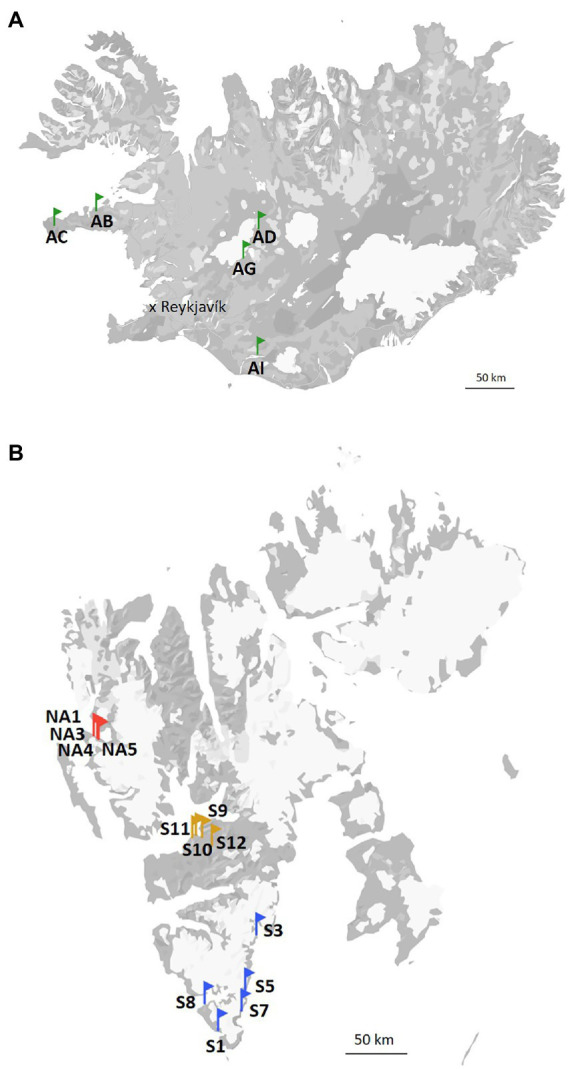
Map of the sampling sites in Iceland **(A)**, and Svalbard **(B)**.

The terrains in Svalbard during the summer period are snow-free, with the melt season starting around end of May or beginning of June. Vegetation consists mainly of lichens and mosses, but vascular plants (e.g., *Salix polaris*, *Dryas octopetala*, *Saxifraga* spp., *Betula nana* etc.) are also sparsely present. Ny-Ålesund is located at the west coast of Svalbard (78°55′26.33” N, 11°55′23.84″ E). The annual average air temperature is −4°C. The coldest and warmest months of the year are March (average air temperature − 12.7°C) and July (average air temperature 4.4°C), respectively. Longyearbyen (78°13′6.92” N, 15°38′55.50″ E) is the capital of Svalbard and, therefore, the most densely populated area there. The annual average air temperature is −4°C. The average air temperature in March is −12.2°C and in July is 7.4°C. At last, the annual average air temperature is −3°C and the mean air temperature of March and July in South Svalbard are −8.7°C and 4.9°C, respectively (data are from the Hornsund weather station; 76°56′59.99” N, 15°45′59.99″ E). Biocrust samples from Svalbard, High Arctic (Ny-Ålesund, Longyearbyen and South Svalbard) were collected in July and August 2021. The sampling sites in the South Svalbard and Longyearbyen were located around the coast and the samples (around 5 mm depth) were collected from the top of solifluction lobes or elevated sea terraces. No visible algal or cyanobacterial biomass was observed on the surface of the collected biocrusts. Biocrusts from Ny-Ålesund were well-developed with presence of moss and lichens. The three field replicates were collected in each selected site and kept frozen until further analyses. The map of the sampling sites is present in Figure 1.

Iceland (64°08′ N, 21°56′ W) is characterized by a subarctic climate with an annual average temperature of 3.1°C. The coldest and warmest months are February and July with mean temperatures of −6.7 and 8.5°C, respectively. The vegetation is represented by shrub birches, moss heaths, marsh grass, and grassland. The soils in this area are Andosols, which have coarse texture, high porosity, low cohesion, and the dispersal of many layers of tephra caused by frequent volcanic eruptions. Andosols tend to accumulate more organic matter than other soil types and, therefore, promote microbial growth. Five different sites were visited and two field replicates of biocrusts were collected at each of them (Figure 1).

### Soil analysis

Field replicates from each site were mixed together and passed through a sieve (2 mm mesh). Chemical analysis was performed according to Czech and European Union standards (ISO 10390, ISO 10523, ČSN EN 27888, ISO 11465, ČSN EN ISO 11732, ČSN EN ISO 13395, and ČSN EN ISO 15681–1). Conductivity (μS cm^−1^) and pH were evaluated in demineralized and distilled water, respectively. The samples were further dried at 105°C to constant weight and then combusted at 450°C. Soil organic matter content (SOM) was calculated as difference between two weights. Total phosphorus (TP) was estimated using spectrometric determination of phosphorus soluble in sodium hydrogen carbonate solution. Mineral nitrogen (N_mineral_) was calculated as a sum of N–NH_4_, N–NO_3_ and N-NO_2_ concentrations, which were measured using a QuikChem^®^8500FIA automated ion analyser (LachatInstruments, Loveland, United States).

### Cell number and biovolume measurements of microbial phototrophs

Number of cyanobacterial and microalgal cells in the biocrusts was counted by light and epifluorescence microscopy (BX51, Olympus) using two technical replicates from each mixed-together sample (as described in soil analysis). A non-staining method was employed using chlorophyll autofluorescence according to (Kaštovská et al., 2005). Biocrust sample (1 g) was suspended in 4 ml of distilled water and mixed thoroughly. A total of 20 μl of this suspension was used for the microscopy. Cyanobacteria and eukaryotic microalgae were quantified using filter cubes (Olympus, MWG) with green excitation at 510–550 nm (emission 590 + nm) and blue excitation at 450–480 nm (emission 515 + nm), respectively. Three groups of cyanobacteria were identified according to their cell morphology: unicellular, filamentous and heterocystous cyanobacteria. Within eukaryotic microalgae, diatoms, unicellular and filamentous microalgae were recognized. Basic geometric equations for cylinders with hemispherical ends and spheres were applied to calculate cell biovolume (Hillebrand et al., 1999).

### DNA isolation and amplicon sequencing

Total DNA was extracted from 98 biocrusts samples (with two technical replicates from each collected field replicate) using the DNeasy PowerSoil Pro Kit (QIAGEN, United States) according to the manufacturer’s instructions. The DNAs were then sent to the Microsynth AG (Balgach, Switzerland), where PCR and sequencing using Illumina MiSeq platform were performed. Two replicates of site S9 was too low and, therefore, were not sent for the sequencing.

Two sets of primers were used for the amplification of V3–V4 region of the 16S rRNA gene and V4 region of the 18S rRNA genes: bacterial (341F – CCTACGGGRSGCAGCAG, 802R – TACNVGGGTATCTAATCC; Watanabe et al., 2001) and eukaryotic (tarEuk_F – CCAGCASCYGCGGTAATTCC, tarEuk_R – ACTTTCGTTCTTGATYRA; Stoeck et al., 2010). The raw reads were submitted to the Sequence Read Archive (SRA) under the project PRJNA881983.

### Bioinformatic and statistical analyses

Obtained demultiplexed Illumina pair-end raw sequences were merged using USEARCH (version 11.0.667; Edgar, 2010) and, subsequently, quality filtered using VSEARCH (version 2.14.1; Rognes et al., 2016). Clustering the reads into amplicon sequence variants (ASVs) using the UNOISE algorithm, their taxonomic assignments and ASV table construction was performed in USEARCH. ASV taxonomic assignments were conducted based on Living Tree Project (LTP; version 123) implemented in SILVA database for 16S rRNA and SILVA database (version 123) for 18S rRNA. ASVs classified as chloroplasts, mitochondria and Archaea were discarded from the bacterial dataset. Additionally, Greengenes database was used separately for the taxonomic assignments of cyanobacterial ASVs.

All statistical analyses were performed in R (version 4.1.3). A few samples, which had very low number of reads, were discarded prior to the analyses (3 and 8 samples from 16S and 18S datasets, respectively) and a minimum of three technical replicates per site remained. Alpha diversity indices (ASV richness, Chao1 and Shannon’s diversity) were calculated using the package *vegan* (Oksanen, 2013). The differences in parameters among sampling sites and regions were tested with one-way analysis of variance (ANOVA) and Tukey’s HSD *post-hoc* test (value of *p* < 0.05). Normality of variance was assessed using Shapiro–Wilk’s test. Community dissimilarities among the sampling sites and four localities obtained by amplicon sequencing were assessed by non-metrical multidimensional scaling (NMDS) based on reads number using the package *vegan*. Additionally, permutational multivariate analysis of variance (PERMANOVA) as well as pairwise multilevel comparison were performed. Soil parameters were fitted into the ordination space using the function *envfit* and significance of the associations was determined by 9,999 random permutations. Pearson correlation test between soil chemistry and diversity indices, number of microphototrophic cells and their biovolume was also performed. Furthermore, Venn diagrams were created for both bacterial and eukaryotic datasets to determine the relations between the sites. In addition, probabilistic co-occurrence analysis was conducted using the package *cooccur* (*true_rand_classifier* = 0.2), which focuses on pairwise comparisons of species. When possible, the lowest taxonomical classification was used for each group of organisms (from order to phylum; Supplementary Table 1).

## Results

### Soil characteristics

Chemical characteristics of the biocrusts are presented in Table 1. In brief, the pH of the biocrusts was in the range of 5.0–7.0 in Iceland, 5.7–7.3 in Ny-Ålesund, 4.7–6.6 in Longyearbyen and 4.8–8.2 in South Svalbard. The highest conductivity was observed in the biocrusts from Ny-Ålesund, while the lowest was reported in the South of Svalbard. SOM did not exceed 10% across all studied biocrusts, except one site in Ny-Ålesund (NA1).

**Table 1:** Soil chemistry of the studied sites.

	Site	pH (H_2_O)	Conductivity, μS/cm	SOM, %	N_mineral_, %	TP, %
Iceland	AB	4.98	83.70	1.34	0.08	0.10
	AC	5.40	46.65	2.96	0.19	0.08
	AD	5.13	87.40	2.43	0.14	0.11
	AG	5.45	29.50	2.04	0.14	0.05
	AI	6.98	98.45	1.72	0.19	0.06
Ny-Ålesund	NA1	7.05	163.33	25.77	2.10	0.11
	NA3	7.26	225.13	1.29	0.20	0.07
	NA4	5.74	97.23	8.95	0.60	0.08
	NA5	7.06	252.17	5.28	0.42	0.07
Longyearbyen	S9	6.64	49.55	4.57	0.09	0.07
	S10	4.68	106.70	5.60	0.10	0.04
	S11	6.12	33.95	3.94	0.13	0.07
	S12	5.34	20.00	5.14	0.15	0.06
South Svalbard	S1	8.22	146.10	2.71	0.07	0.05
	S3	5.87	10.35	4.95	0.08	0.05
	S5	4.77	18.10	3.84	0.07	0.04
	S7	6.29	16.15	4.18	0.12	0.08
	S8	8.06	62.85	2.44	0.07	0.02

### Cells number and biovolume of microbial phototrophs

**Figure 2:**
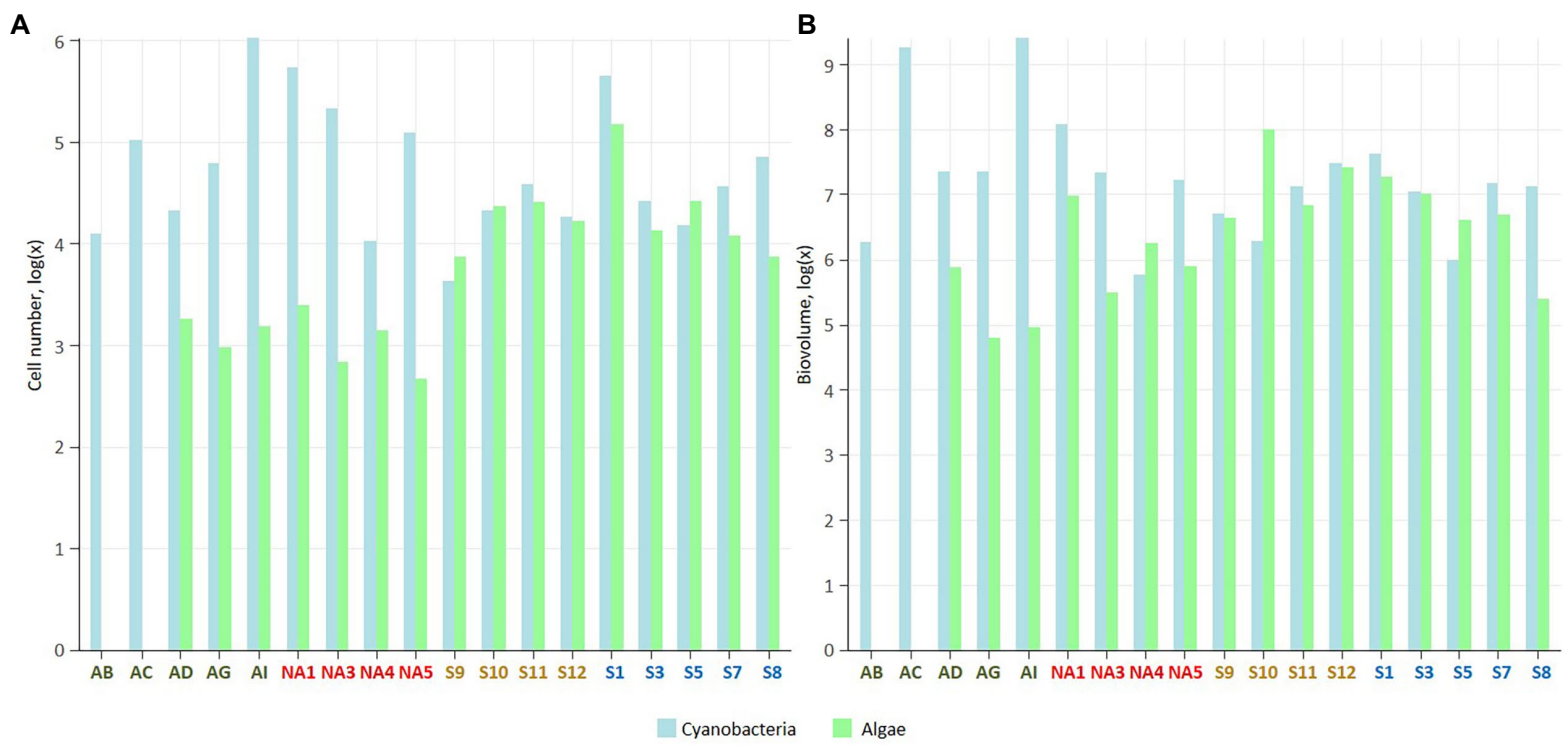
Number of cells **(A)**, and cell biovolume (**B**; μm^3^ g^−1^ dry soil) in the studied biocrusts. The samples colour codes: green – Iceland, red – Ny-Ålesund, brown – Longyearbyen, blue – South Svalbard. The data were log transformed.

Number of cells and biovolume of microbial phototrophs measured using epifluorescence microscopy varied among the sites and localities (Figure 2; Supplementary Table 2). However, the differences among the localities were not significant as biocrusts were very heterogeneous and abundance varied depending on the site.

Average number of cells and biovolume of cyanobacteria was the highest in biocrusts from Iceland (25.5 × 10^4^ cells g^−1^ and 887.5 × 10^6^ μm^3^ g^−1^, respectively) and Ny-Ålesund (22.6 × 10^4^ cells g^−1^ and 39.1 × 10^6^ μm^3^ g^−1^, respectively), with the dominance of heterocystous and filamentous cyanobacteria, respectively. The lowest cyanobacterial measurements were observed in the biocrusts from Longyearbyen (2.1 × 10^4^ cells g^−1^ and biovolume of 12.6 × 10^6^ μm^3^ g^−1^). Furthermore, average cell number of eukaryotic microalgae was the highest in the South Svalbard (4.5 × 10^4^ cells g^−1^) and the lowest in Iceland (0.1 × 10^4^ cells g^−1^). Biocrusts from Iceland and Longyearbyen were dominated by diatoms within eukaryotic microalgal community, while green and yellow-green coccal microalgae prevailed in other biocrusts.

Cyanobacterial number of cells exceeded microalgal ones in all four localities and the highest cyanobacteria/microalgae ratio was observed in Icelandic biocrusts (cyanobacteria/microalgae = 296/1; Figure 2; Supplementary Table 2). Biovolume cyanobacteria/microalgae ratios showed similar trends except Longyearbyen samples, where microalgal biovolume surpassed cyanobacterial (cyanobacteria/microalgae = 1/3; Figure 2; Supplementary Table 2).

### Microbial community composition

**Figure 3:**
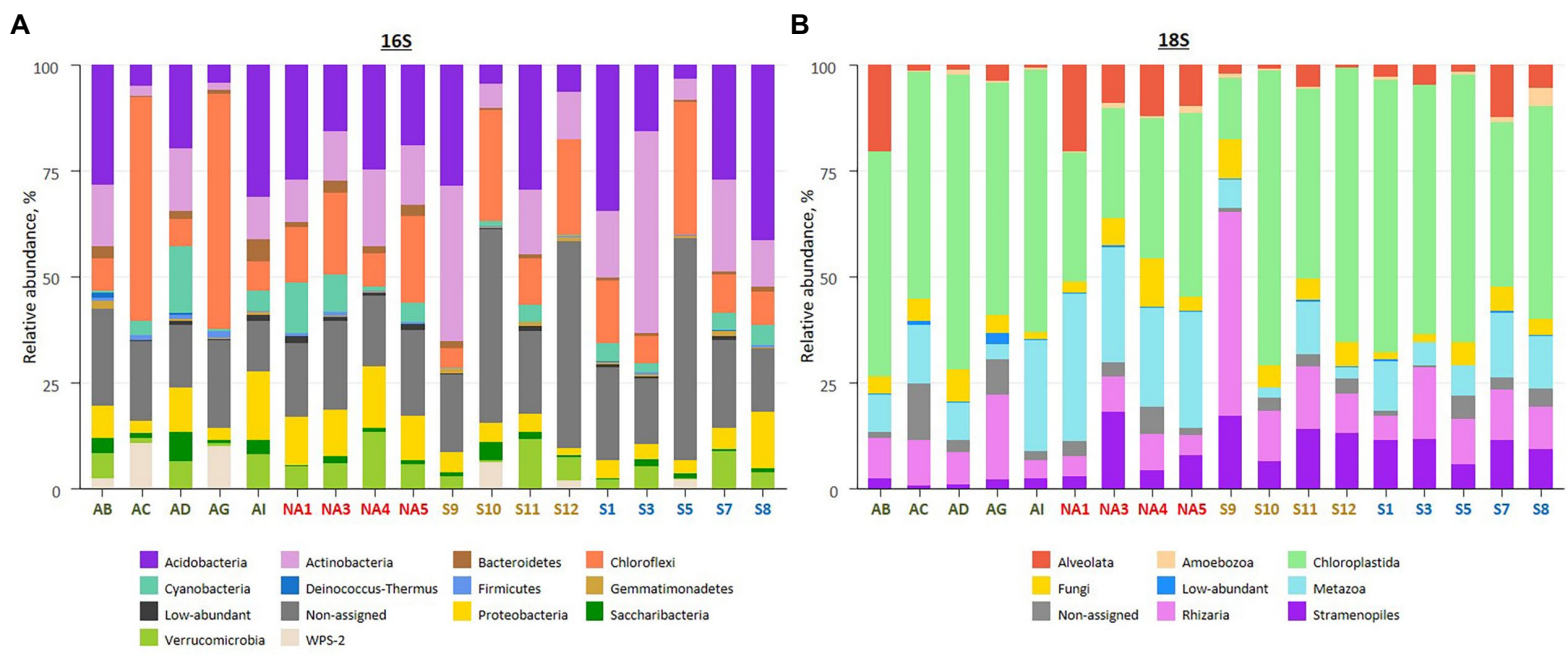
Overview on biocrust microbial community composition in the studied localities based on relative abundances of bacterial phyla **(A)**, and eukaryotic clades **(B)**. The samples colour codes: green – Iceland, red – Ny-Ålesund, brown – Longyearbyen, blue – South Svalbard. Low-abundant combines phyla or clades with relative abundance <0.5%. Each bar represents an average of replicates.

Amplicon sequencing using general bacterial primers resulted in 2 M quality filtered reads, clustered into 13,762 ASVs. Of this, 10,793 ASVs were assigned to phyla and 2,969 ASVs (25% of total reads) assigned only to the kingdom (non-assigned bacteria). Taxonomic assignment of obtained ASVs revealed the dominance of Acidobacteria (2,763 ASVs, 19.3% of total reads), Actinobacteria (2031 ASVs, 12.7% of total reads) and Chloroflexi (869 ASVs, 18.4% of total reads) across all samples (Figure 3A; Supplementary Tables 1, 3). Biocrusts in Ny-Ålesund and South Svalbard had higher percentage of Acidobacteria (21.7 and 25.5% of reads, respectively), while biocrusts from Iceland and Longyearbyen were dominated by Chloroflexi (25.8 and 17.7% of reads, respectively).

Amplicon sequencing of 18S rRNA revealed 5 M quality filtered reads and 6,909 ASVs. The majority of ASVs belonged either to Chloroplastida (652 ASVs, 50.1% of total reads), Metazoa (707 ASVs, 15.0% of total reads) or Rhizaria (1787 ASVs, 10.8% of total reads; Figure 3B; Supplementary Tables 1, 3). In addition, 765 ASVs (3.7% of total reads) were not assigned to any phyla. The majority of Chloroplastida reads belonged to Embryophyta (39.0, 23.7, 18.3 and 21.9% of reads in Iceland, Ny-Ålesund, Longyearbyen and South Svalbard, respectively) and Trebouxiophyceae (13.9, 5.3, 25.1 and 21.4% of reads in Iceland, Ny-Ålesund, Longyearbyen and South Svalbard, respectively). Moreover, Ulvophyceae abundance was significantly higher in biocrusts from South Svalbard than in other localities (3.8% of reads). Furthermore, there were significant differences in Metazoa abundances among the localities. The highest number of Metazoa reads was recorded in Ny-Ålesund (28.1% of reads) with the dominance of phylum Nematoda (19.9% of reads). Besides, Gastrotricha, observed in Ny-Ålesund biocrusts, was not reported in other localities. In addition, Rhizaria, represented by only phylum Cercozoa, had significantly higher relative abundance in biocrusts from Longyearbyen (17.0% of reads) than in the other localities. At last, within Ochrophyta community, Bacillariophyceae were significantly more abundant in biocrusts from Longyearbyen (5.9% of reads), while Xanthophyceae prevailed in biocrusts from South Svalbard (5.8% of reads).

**Figure 4:**
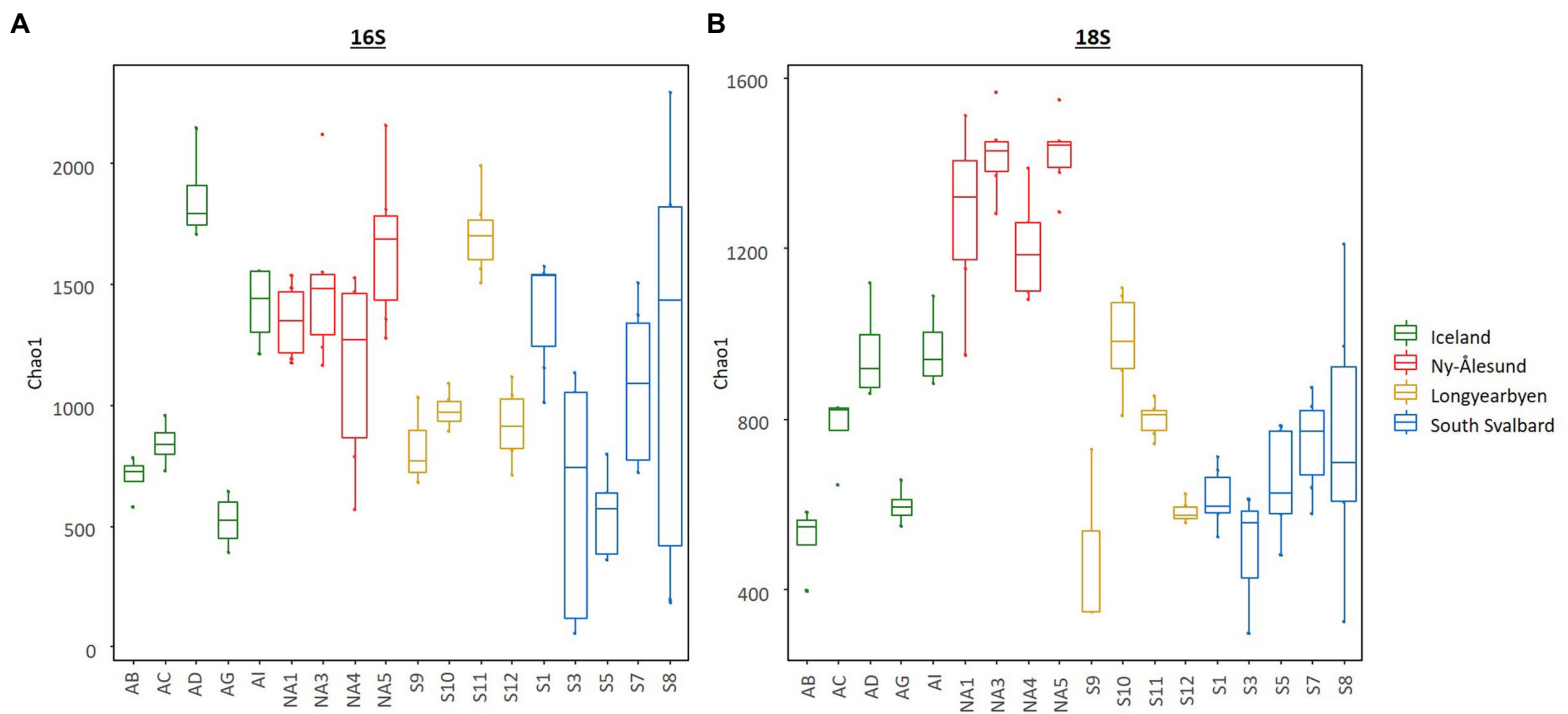
Box plots illustrating Chao1 diversity index in the biocrusts from the studied sites. **(A)** – bacteria and **(B)** – eukaryotes. Boxes represent the interquartile range and the horizontal line inside the box defines the median.

**Figure 5:**
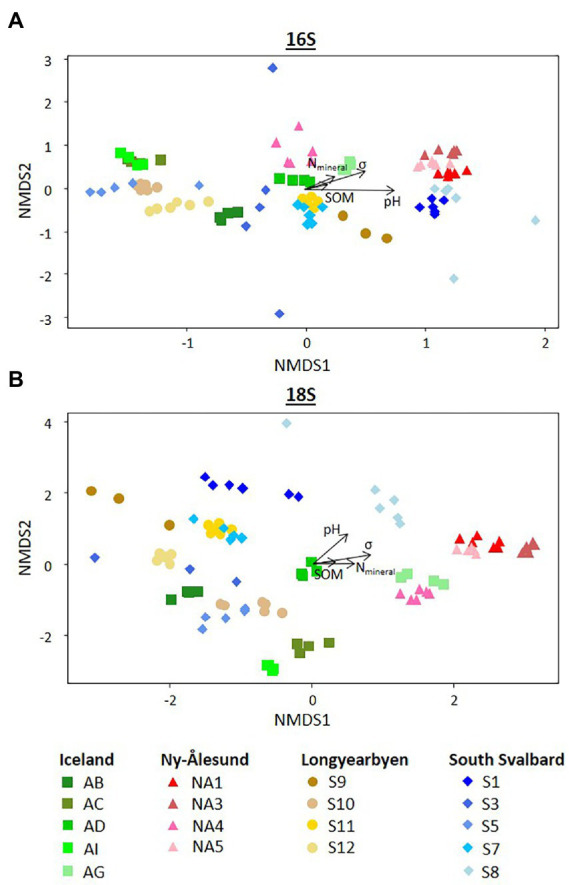
Non-metric multidimensional scaling (NMDS) plots based on the ASVs abundance (number of reads) of **(A)** 16S and **(B)** 18S rRNA genes. Arrows indicate significant correlations (*p* < 0.05) with environmental variables. SOM soil organic matter, N_min_ – mineral nitrogen, σ – conductivity.

Diversity indices calculated for bacteria and eukaryotes overall differed between the sites and were higher in the biocrusts from Ny-Ålesund (Figure 4, only Chao1 is shown as an example; Supplementary Table 4). However, large variations between sites within each locality were observed. Non-metric multidimensional scaling plot based on ASV abundance showed that microbial communities were distinct among the sites, but the individual replicates grouped by the site of origin (Figure 5). Besides, PERMANOVA confirmed that the differences between the localities were significant (Supplementary Table 5). However, the r^2^ was very low meaning that there were a lot of unexplained variations in these models. In addition, pH, conductivity, SOM and N_mineral_ had significant effect on microbial community composition (Figure 5). Moreover, Pearson correlation test revealed that diversity indices were significant positively correlated with conductivity, pH (only Chao1 for 16S) and N_mineral_ (only for 18S; Table 2). Besides, significant positive correlations were observed between pH and cyanobacterial number of cells.

**Table 2:** Correlations between soil chemical parameters and diversity and abundance measurements assessed by Pearson correlation test.

	Number of cells		Biovolume		16S diversity indices		18S diversity indices	
	Cyanobacteria	Microalgae	Cyanobacteria	Microalgae	Richness	Chao1	Richness	Chao1
pH	0.50*	0.37	0.07	−0.30	0.47	0.47*	0.30	0.29
Conductivity	0.35	0.08	−0.03	0.02	0.6**	0.59**	0.81***	0.78***
SOM	0.21	−0.11	−0.15	0.09	0.10	0.10	0.37	0.39
N_mineral_	0.34	−0.17	−0.03	−0.06	0.21	0.21	0.50*	0.52*
TP	0.05	−0.29	0.03	−0.31	0.31	0.33	0.23	0.25

´–´ correspond to negative correlations, while no sign indicates positive correlations. Significant correlations are marked in bold (* *p* < 0.05, **p < 0.01, ***p < 0.001).

**Figure 6:**
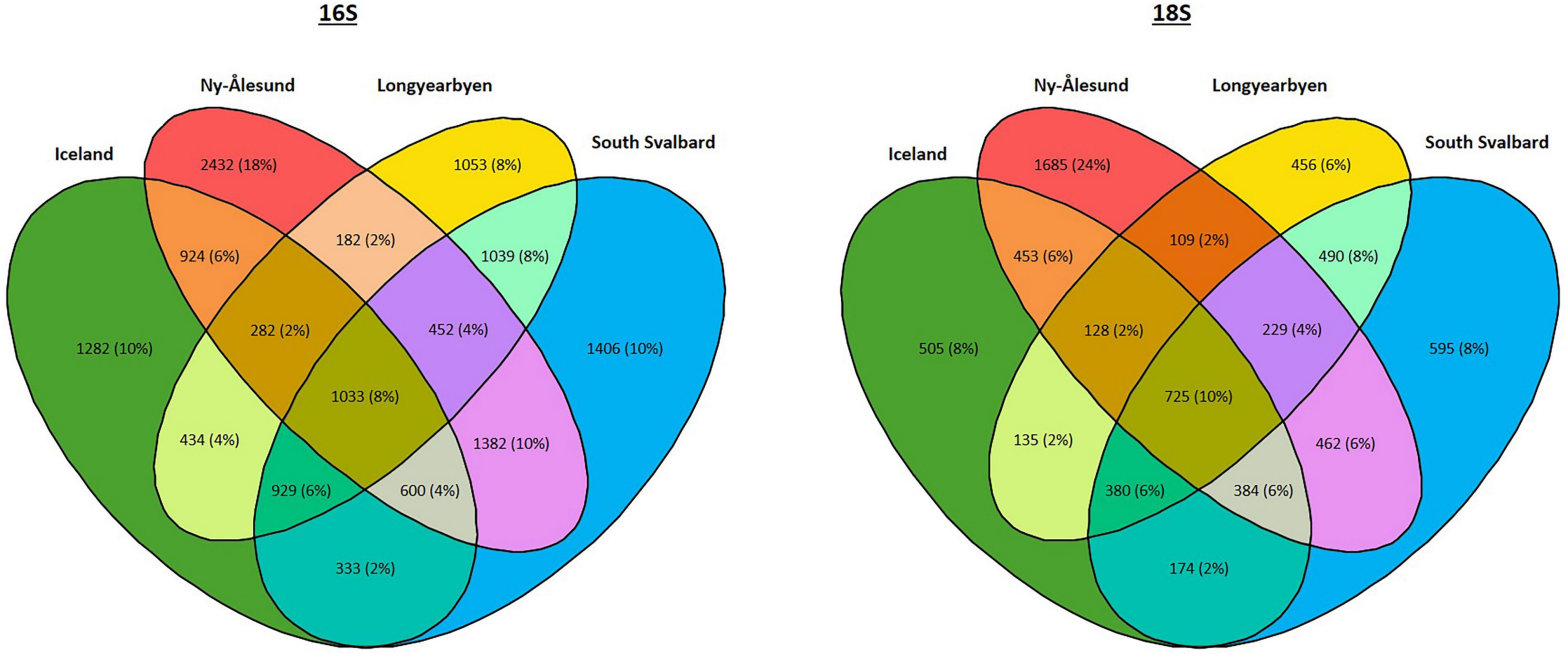
Venn diagram showing the number of common ASVs among the localities.

Furthermore, 8 and 10% of bacterial and eukaryotic ASVs, respectively, were found in biocrusts from four localities (Figure 6, Supplementary Table 6). The highest percentage of ASVs (25% of prokaryotic and 26% of eukaryotic ASVs) were shared between South Svalbard and Ny-Ålesund and the same numbers between South Svalbard and Longyearbyen. Ny-Ålesund and Longyearbyen had the lowest number of common ASVs (14% of prokaryotic and 17% of eukaryotic ASVs). The highest number of unique ASVs were observed in Ny-Ålesund (18% of prokaryotic and 24% of eukaryotic ASVs).

**Figure 7:**
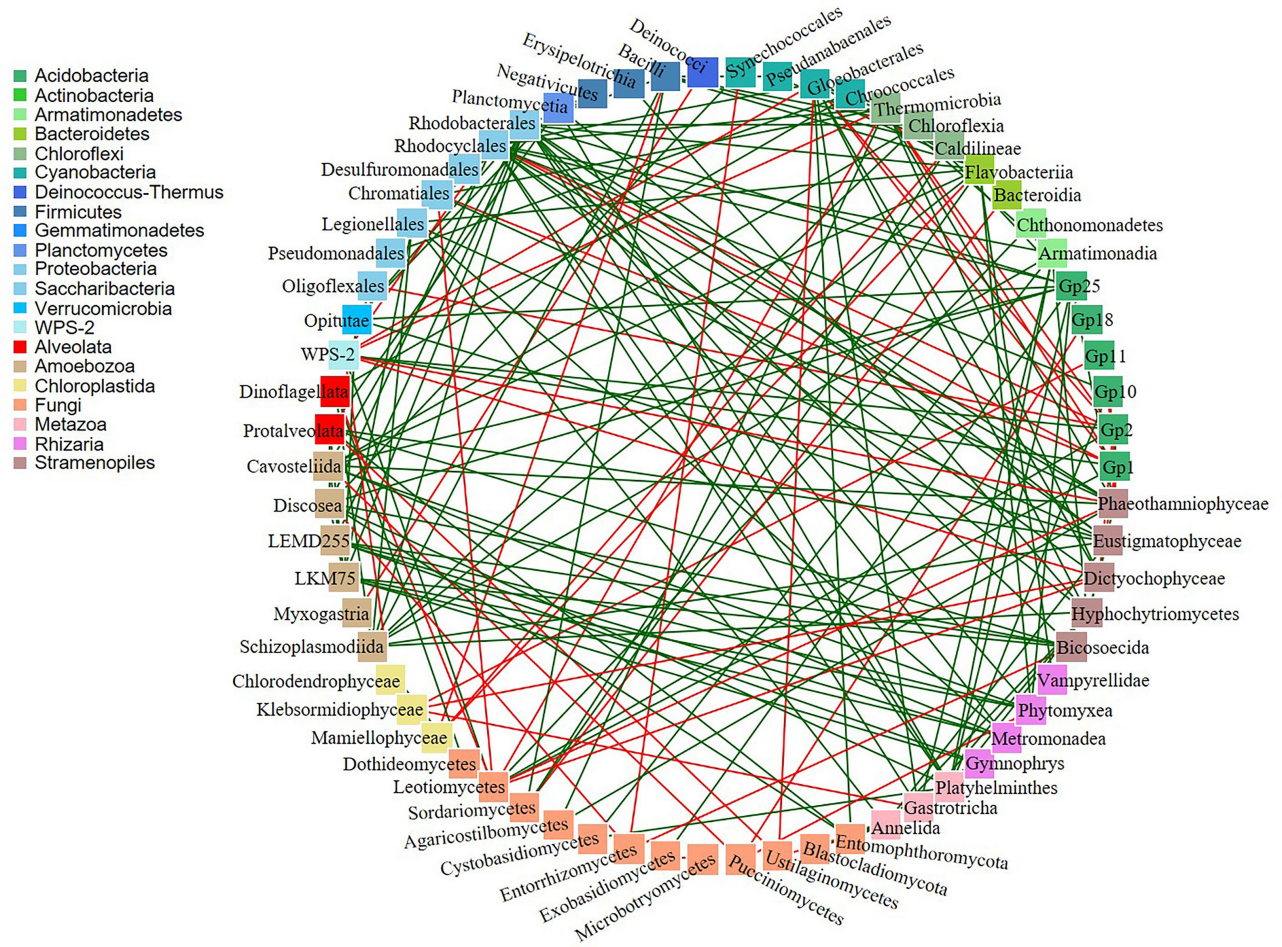
Co-occurrence networks of bacteria and eukaryotes. Green and red lines indicate positive and negative correlations, respectively.

In addition, co-occurrence networks revealed 188 significant potential interactions in the biocrusts (Figure 7; Supplementary Table 7). Of these, 143 interactions were positive and 45 were negative. The majority of both positive and negative interactions were between bacteria and eukaryotes (18 negative and 63 positive). Out of 137 microbial taxa, 72 taxa did not show any co-occurrence.

## Discussion

### Microbial community composition in arctic and sub-arctic regions

In general, community compositions and abundance of prokaryotes and eukaryotes significantly differed among the biocrusts (Figures 2–5; Supplementary Tables 1–4). The average of diversity indices in biocrusts from Ny-Ålesund were higher than in the other localities. Together with high abundance and biovolume, this could indicate richer biocrust microbial communities there. Biocrusts cover up to 90% of the Ny-Ålesund area and higher water availability promotes the growth of biocrust microbiota (Williams et al., 2017).

Microbial community structure in the biocrusts assessed by amplicon sequencing was overall similar to those previously studied in Svalbard and Iceland (Rippin et al., 2018b; Pushkareva et al., 2021a), but with the dominance of Acidobacteria, Chloroflexi and Actinobacteria within prokaryotes. These bacteria are very common in the north soil ecosystem (Malard and Pearce, 2018; Pushkareva et al., 2021a), but, so far, was never reported to dominate biocrusts in Svalbard and Iceland. Within Chloroflexi, class Ktedonobacteria dominated Icelandic biocrusts (79% of Chloroflexi reads). These aerobic bacteria form branched mycelia with spores and favour extreme volcanic environments (Tebo et al., 2015). However, functions of Ktedonobacteria remain unclear (Zheng et al., 2019). The majority of Chloroflexi sequences in biocrusts from Ny-Ålesund belonged to class Caldilineae (63% of Chloroflexi reads), which was also observed in the other studied localities with lower number of reads. Members of Caldilineae are non-sporulating filamentous bacteria favouring neutral pH (Sekiguchi and Hanada, 2020). Furthermore, genomic studies of Acidobacteria showed their involvement in carbon, nitrogen and sulfur cycles, which is important in arctic and sub-arctic terrestrial environments (Kalam et al., 2020). Besides, Acidobacteria are oligotrophs and produce extracellular polymeric substances (EPS), which contribute to their life in extreme environments and, in turn, EPS might facilitate the formation of biocrusts (Weber et al., 2022). In addition, Actinobacteria are capable to fix nitrogen (Sellstedt and Richau, 2013) and, especially important in polar biocrusts with nitrogen deficiency.

Furthermore, nitrogen-fixing cyanobacteria Nostocales were present in all sites, but had higher biovolume, number of cells and reads in Icelandic biocrusts. These cyanobacteria are capable to form symbioses with different organisms and lichen-associated *Nostoc* sp. isolated from Iceland has been previously described (Gagunashvili and Andrésson, 2018). Likewise, in our study, the majority of ASVs identified to the genus level from the order Nostocales corresponded to Nostoc. Number of cells and biovolume of cyanobacteria were higher than of eukaryotic microalgae in all three localities and the highest cyanobacteria / microalgae ratio was observed in Icelandic biocrusts. Cyanobacteria are primary producers and considered as biocrusts engineers (Barrera et al., 2022). On contrary, the majority of eukaryotic microalgae algae do not actively participate in biocrust formation but rather establish associations with other biocrust organisms (Büdel et al., 2016).

The dominance of Chloroplastida in the biocrusts recorded with amplicon sequencing, was in agreement with other studies from these regions (Rippin et al., 2018b; Pushkareva et al., 2021a). Biocrusts from Iceland had significantly higher abundance of Embryophyta than in the other localities. Large territories of this island are represented by tundra with moss being the main vegetation. Likewise, Ny-Ålesund is surrounded by Arctic tundra and high percentage of Embryophyta in the biocrusts is not surprising. Furthermore, biocrusts from Longyearbyen and South Svalbard had significantly higher number of reads and cells assigned to Trebouxiophyceae, Chlorophyceae, Zygnematophyceae and Xanthophyceae than in the other two localities. Diatoms abundance of reads and cells were significantly higher in biocrusts from Longyearbyen. Eukaryotic microalgae are typical inhabitants of polar biocrusts and have previously been recorded to be more abundant in biocrusts from Longyearbyen than Ny-Ålesund (Rippin et al., 2018b). Furthermore, Thecofilosea (Cercozoa, Rhizaria), significantly more abundant in biocrusts from Longyearbeyen, feeds mainly on algae, which could explain their dominance in this locality. Bacterivorous order Glissomonadida, in particular genus *Heteromita*, was abundant in all studied localities and it is well known inhabitant of various biocrust types (Roshan et al., 2021).

Ascomycota, Basidiomycota and Chytridiomycota dominated the biocrusts fungal community. Several members of the first two phyla are known to produce antifreeze proteins (AFPs) as well as protective sugars, lipids, polyols, and fatty acids which promote their growth in cold environments (Robinson, 2001). Chytridiomycota parasitize cyanobacteria and microalgae (Gleason et al., 2008), but little is known about their survival mechanisms in polar biocrusts. A study from Australia showed that these fungi can resist periodic drying and high temperatures (Gleason et al., 2004). Furthermore, family Verrucariaceae (Ascomycota) were the only lichenised fungi in the biocrusts and had higher abundance in biocrusts from South Svalbard and Longyearbyen. Besides, lichens Verrucaria were previously recorded in Svalbard (Wietrzyk-Pełka et al., 2018).

There was a high number of Metazoa reads in the studied biocrusts with significant prevalence in Ny-Ålesund. Among soil fauna, Nematoda are one of the most abundant groups in soil ecosystem (Yeates and Bongers, 1999) and also was the main representative of Metazoa in the studied biocrusts. Positive relations between biocrusts and nematodes communities have been previously described and, besides, higher abundance of nematodes indicates more developed biocrusts with higher organic matter content (Liu et al., 2011).

In addition, the environmental vector fitting analysis indicated that soil parameters such as pH, conductivity, SOM and N_mineral_ contents significantly influenced the community composition of both bacteria and eukaryotes (Table 2; Figure 5). These soil parameters are well known determinants of microbial community composition across various landscapes (Lauber et al., 2009). Furthermore, Pearson correlation test showed the positive effect of conductivity on the alpha diversity indices. Conductivity has been previously shown to promote bacteria growth (Canfora et al., 2014).

### Potential interactions of microorganisms in arctic and sub-arctic biocrusts

Co-occurrence networks are often used to discover statistically significant associations among microbial communities. There were positive and negative potential microbial interactions in the studied biocrusts (143 and 45, respectively; Figure 7; Supplementary Table 7). The dominance of positive correlations could indicate the occurrence of a mutualistic relations between the species (Steele et al., 2011). On contrary, presence of negative correlations could suggest either competition or predation relationship among organisms (Faust and Raes, 2012).

Fungi can develop a wide range of interactions with bacteria, either beneficial or detrimental (Deveau et al., 2018). The negative Bacteria/Fungi potential interactions revealed by co-occurrence network analysis might suggest that bacteria and fungi in polar and sub-polar biocrusts acquire antagonistic strategies to compete for the derived substrates (de Boer et al., 2005). On the other hand, some bacteria live on or inside fungal cells and exploit fungal-secreted metabolites (Stopnisek et al., 2016) resulting in positive mutualistic relationships. Furthermore, the positive potential co-occurrences of different bacterial taxa could indicate their partnerships in the biogeochemical cycles. For example, phototrophic cyanobacteria produce soil organic matter which is further used by heterotrophic bacteria as a source of carbon and/or energy. In this respect, there were six positive potential interactions between these organisms in the studied samples (Supplementary Table 7).

Biocrusts consist of diverse community of Metazoa and Protozoa, which feed mainly on bacteria, but also on algae (Potapov et al., 2022). In this regard, negative correlations of Myxogastria (Amoboezoa)/Deinococci (Bacteria) and Gastrotricha (Metazoa)/Klebsormidiophyceae (Chloroplastida) were observed in the biocrusts. There are no studies about Gastrotricha feeding on Klebsormidiophyceae, although some showed that they can digest green alga *Chlorella*, suggesting that microalgae are of secondary importance in Gastrotricha diets (Strayer et al., 2010). Furthermore, some protists are able to form different types of symbiosis with fungi, other protists, plants and small animals (Geisen et al., 2018). There were 40 positive correlations among these organisms, indicating high probability of symbiotic relationships in the studied biocrusts.

However, co-occurrences may not only reflect shared niche preferences and relationships among biocrusts microorganisms but also could be a result of random mixing within the microbial community. Besides, some microbial relationships could be overlooked. For example, lichens are frequently observed in polar biocrusts and were also present in the studied biocrusts. *Trebouxia* is the most common lichen photobiont (Friedl and Rybalka, 2012), but there were no potential interactions between Trebouxiophyceae and fungi recorded by co-occurrence analysis.

## Conclusion

This study provided a description and comparison of biocrusts from Svalbard and Iceland. Biocrusts from South Svalbard had overall the least diverse microbial community composition in comparison to the other localities. On contrary, Iceland and Ny-Ålesund both exhibited high diversity and abundance of biocrust microbiota, but had different community structure. There was no trend in microbial diversity following the latitude gradient. However, biocrusts community composition were influenced by conductivity, pH, soil organic matter and mineral nitrogen contents. Furthermore, targeting both 16S and 18S rRNA allowed understanding of potential interactions among different taxa. In this regard, there were more positive bacteria/eukaryotes interactions suggesting that these organisms promote each other’s growth by increasing nutrient availability and creating new niches.

Contributing to the knowledge about diversity and abundance of biocrusts microbiota and potential interactions between various species could be a key aspect to understanding the dynamics of ecosystems, especially in the arctic and sub-arctic environments.

## Data availability statement

The data presented in the study are deposited in the Sequence Read Archive (SRA), accession number PRJNA881983.

## Author contributions

EP and BB designed the study. JE, AH and SN collected the samples. JE provided soil chemistry and microscopy measurements. EP performed the molecular work, processed the data, and wrote the manuscript. BB, AH and JE performed the revision. All authors contributed to the article and approved the submitted version.

## Funding

EP and BB were supported by the Deutsche Forschungsgemeinschaft (DFG) within the (project BE1779/23-1) which is part of the Priority Program 1158 ‘Antarctic Research’. JE was funded by the Ministry of Education, Youth and Sport of the Czech Republic [projects LTAIN 19139]; by the Czech Science foundation [project 22-08680 l] and by the Czech Academy of Sciences [long-term research development project No. RVO 67985939]. AH was supported by Austrian Science Fund (FWF) grant P34181-B.

## Conflict of interest

The authors declare that the research was conducted in the absence of any commercial or financial relationships that could be construed as a potential conflict of interest.

## Publisher’s note

All claims expressed in this article are solely those of the authors and do not necessarily represent those of their affiliated organizations, or those of the publisher, the editors and the reviewers. Any product that may be evaluated in this article, or claim that may be made by its manufacturer, is not guaranteed or endorsed by the publisher.
